# Ras Homolog Enriched in Brain Protein Reverses Amyloid Beta‐Induced Escape of Inflammatory Cytokine mRNAs From Immunoisolated RNA Processing Bodies of Glioblastoma Cells

**DOI:** 10.1096/fj.202502872R

**Published:** 2025-12-13

**Authors:** Sritama Ray, Kamalika Mukherjee, Suvendra N. Bhattacharyya

**Affiliations:** ^1^ CSIR‐Indian Institute of Chemical Biology Kolkata West Bengal India; ^2^ Department of Anesthesiology University of Nebraska Medical Center Omaha Nebraska USA; ^3^ Department of Pharmacology and Experimental Neuroscience University of Nebraska Medical Center Omaha Nebraska USA

**Keywords:** amyloid beta, cytokine expression, mRNA, neuroinflammation, phase‐separated structures, RNA processing bodies

## Abstract

Post‐transcriptional regulation by RNA processing bodies, also known as P‐bodies (PBs), is vital for mRNA translation, localization, and stability in various animal cells, including neurons and glial cells. PBs enable spatial control of protein synthesis, influencing differentiation and synaptic function. Understanding mRNA storage within PBs is essential for identifying the mechanism controlling post‐transcriptional gene expression in brain cells and for understanding its role in neurodegenerative diseases. We developed a detergent‐based method to isolate phase‐separated P‐bodies, free of cytosolic factors and RNAs, enabling us to analyze the mRNA content of PBs under different cellular or experimental conditions. Using neuronal (NGF‐differentiated PC12) and glial cell (C6 glioblastoma) models, we studied how Aβ_1–42_‐oligomers affect PB‐associated mRNAs. In neuronal cells, exposure to Aβ_1–42_‐oligomers disrupted the release of mRNAs associated with neuronal differentiation, impairing their translation. Whereas astroglial cells showed increased levels of cytoplasmic pro‐inflammatory cytokine mRNAs after amyloid treatment, these mRNAs escaped from RNA processing bodies, leading to enhanced translation. Interestingly, ectopic expression of Ras Homolog Enriched in Brain (Rheb) protein, known to influence miRNA reactivation, helped restore the localization of inflammatory cytokine mRNAs IL‐6 and IL‐1β to PBs and lowered their expression in Rheb‐activated astroglia. Our findings suggest that targeting cytokine mRNAs to PBs could be a potential strategy to manage inflammation in activated astroglia in neurodegenerative diseases. At the same time, PB isolation from detergent‐permeabilized cells can be an effective, simplified method for studying PB‐RNA dynamics in eukaryotic cells.

## Graphical abstract

A detergent‐based method, combined with immunoprecipitation using an anti‐P‐body component‐specific antibody, was used to isolate RNAs associated with RNA processing bodies (PBs) from both neuronal and glial cells. In neurons, β‐amyloid oligomers helped to trap specific mRNAs related to neuronal differentiation into PBs. Meanwhile, in astroglia, β‐amyloid induced the release of mRNAs encoding inflammatory cytokines from PBs. When the Ras homolog enriched in brain (Rheb) protein was expressed ectopically, it restored the targeting of cytokine mRNAs to PBs, highlighting its important role in keeping inflammatory cytokines repressed in glial cells affected by amyloid proteins.

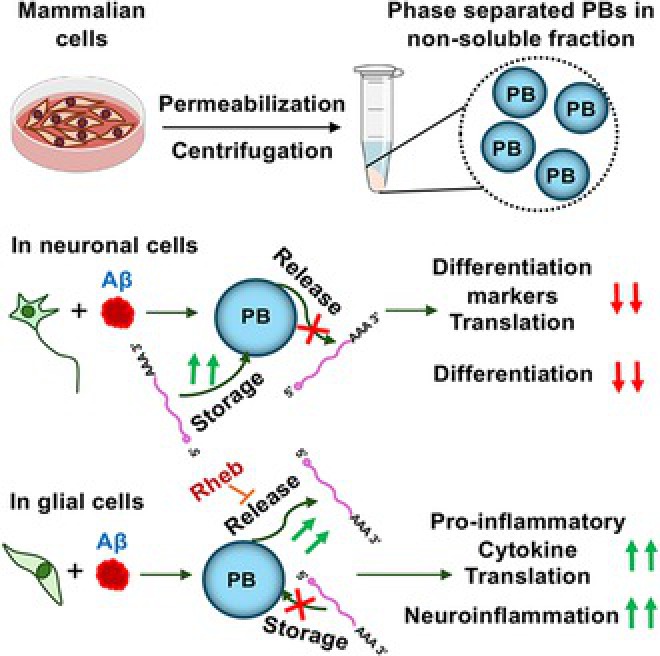

## 1 Introduction

RNA regulation at the pre‐ and post‐transcriptional levels is influenced by multiple factors that orchestrate protein expression at specific times and in particular compartments in metazoan cells. This spatiotemporal control of protein synthesis is governed by the accessibility of mRNAs encoding the protein to translational machineries, which are regulated by various RNA regulatory factors that bind to specific sequences or motifs of the mRNAs through direct or indirect (miRNA‐mediated) interactions [1, 2]. These interactions control translatability, storage, and degradation of mRNAs. RNA‐protein interactions are often linked to the formation of phase‐separated structures involving complex biophysical and biochemical interactions that result in phase‐separated mRNAs, which exhibit reduced accessibility for translating polysomes and the translational machinery [3, 4]. This process facilitates mRNA storage for specific cellular needs or degradation upon miRNA repression in diverse mammalian cell types, including neurons [5, 6].

Among the many regulatory factors involved, microRNAs (miRNAs) play a central role. These small RNA molecules regulate gene expression by binding to complementary regions of target messenger RNAs (mRNAs) [7]. This interaction can either promote the degradation of mRNA or render it translationally inactive, depending on the degree of complementarity between the miRNA and its target sequence, leading either to the storage of target mRNA in a translationally inactive state within specialized phase‐separated structures in the mammalian cells or to its degradation [1, 8, 9, 10].

A critical element of this regulation is RNA processing bodies (PBs), also known as P‐bodies. These cytoplasmic, membrane‐less RNA‐protein condensates form via liquid–liquid phase separation [11]. PBs function as storage hubs for mRNAs bound by miRNA‐induced silencing complexes (miRISC), leading to the translational repression of these mRNAs [12, 13]. Importantly, PBs are dynamic structures rather than static ones, with mRNAs constantly shuttling between these bodies, where mRNAs are stored in an inactive form or getting degraded, and polysomes, where mRNAs are actively translated into proteins. The movement of mRNAs between these two compartments is sensitive to changes in the cellular environment, such as those caused by cellular stress or other extrinsic and intrinsic signals, which can directly impact the regulation of mRNA translation and the cellular protein synthesis machinery [14].

This dynamic regulation of mRNA translation and localization is particularly critical for neurons. Neurons with specialized morphological structures need the regulation of protein synthesis at specific locations within the cell. The localized translation of proteins in particular regions of the neuron, such as dendrites or axonal terminals, is crucial for maintaining synaptic function and plasticity [15]. In neurons, many proteins required for synaptic function are not synthesized in the cell body, where they remain translationally repressed within PBs. These mRNAs are then transported to distal dendritic regions, where they are released from repression and translated into the proteins necessary for synaptic activities [5, 16]. This spatiotemporal control of mRNA translation prevents unnecessary (and potentially harmful) protein accumulation in areas where they are not needed, thereby optimizing neuronal function and maintaining cellular homeostasis.

However, the processes regulating the localization of mRNAs to PBs or translating polysomes are responsive to environmental cues, including neuronal activity and stress factors [1, 5]. Environmental changes can impact cellular homeostasis, and disruptions in these regulatory processes may contribute to the development of disease. Despite substantial progress in understanding mRNA localization and translation regulation, the mechanisms driving differential mRNA localization to PBs and the factors influencing this process remain active research areas over the last two decades. In this context, our study develops an assay system that employs detergent‐based separation of cytosolic content to isolate the insoluble fraction containing phase‐separated structures, including PBs, followed by immunoisolation of PBs and thereby enabling analysis of the mRNA content of PBs across various cell types under different cellular environments. This strategy has been found particularly valuable for studying PB‐localization of mRNAs involved in neuronal differentiation and for identifying extrinsic factors that influence mRNA localization to PBs.

Amyloid Aβ_1–42_ peptides form oligomers that cause neurodegeneration [17]. Amyloid oligomers, linked to neurodegenerative diseases like Alzheimer's Disease (AD), also disrupt miRNA function, impairing gene expression regulation. This dysfunction leads to the expression of pro‐inflammatory cytokines in astroglial cells, contributing to the inflammation observed in AD [18]. How do PBs regulate the protein translation process in astroglial cells or in neurons? Our assays showed that amyloid oligomers cause retention of pro‐inflammatory cytokine mRNAs, such as IL‐6 and IL‐1β, in polysomes, ensuring protein expression by preventing their entry into PBs for degradation. This is likely achieved by inhibiting their interaction with repressing miRNA‐Ago protein complexes (miRNPs), which remain associated with PBs after Aβ_1–42_ oligomers treatment [18]. Impaired miRNP‐mRNA interaction in amyloid‐treated cells leads to target cytokine mRNA accumulation with polysomes, expression of cytokine proteins, and promotes the inflammatory response observed in neurodegenerative diseases [18, 19]. Interestingly, the expression of mTORC1 activator protein Rheb‐Myc ectopically in astroglial cells was found to rescue the miRNAs from P‐bodies, promote mRNA‐miRNA interactions, and increase PB‐localization of repressed mRNAs to induce a decrease in cytokine expression in Aβ_1–42_ oligomers‐treated astroglial cells.

Notably, in NGF‐differentiated rat pheochromocytoma PC12 cells, a model for studying Aβ_1–42_'s effects on sympathetic neurons [20, 21], Aβ_1–42_ oligomer treatment has resulted in altered PB‐localization of mRNAs encoding neuronal growth and differentiation‐controlling proteins. We found that with NGF‐induced differentiation, mRNAs vital for neuronal differentiation, like GAP43, Neurofilament‐M, and HuD, are targeted to PBs in differentiated PC12 cells exposed to Aβ_1–42_ oligomers. In the absence of Aβ_1–42_ oligomers, these mRNAs are not targeted to PBs in differentiating PC12 cells.

This study highlights the intricate relationship between mRNA localization to P‐bodies and post‐transcriptional regulation during neuronal differentiation of PC12 cells and during neuroinflammatory responses triggered by pathogenic proteins in C6 glioblastoma cells. The findings highlight the role of amyloid oligomers in disrupting the regulatory processes of mRNA targeting to PBs, providing deeper insight into how dysregulation of RNA processing contributes to disease pathology. Our research offers new insights into potential therapeutic strategies targeting RNA regulation, with significant implications for diseases characterized by disrupted RNA homeostasis, such as neurodegenerative disorders (e.g., Alzheimer's disease) and chronic inflammatory conditions. Future research in this area will further elucidate the mechanisms governing mRNA localization and translation, offering potential pathways for developing novel treatments for these challenging diseases. The method described here for studying PB‐associated mRNAs is qualitative and, more importantly, essential for revealing the mechanism by which amyloid proteins control phase separation of mRNAs in neuronal and non‐neuronal cells.

## 2 Materials and Methods

### 2.1 Cells and Culture Conditions

PC12 cells were cultured in Dulbecco's modified Eagle's medium (DMEM; GIBCO) supplemented with 10% heat‐inactivated horse serum (HS; GIBCO), 5% heat‐inactivated fetal bovine serum (FBS; GIBCO), and 1% Penicillin–Streptomycin. PC12 cells were differentiated by using 100 ng/mL NGF (Promega) added in differentiation medium containing DMEM (GIBCO) supplemented with 0.75% heat‐inactivated HS (GIBCO) and 0.25% heat‐inactivated FBS (GIBCO) for 72 h. C6 cells were cultured in DMEM (GIBCO) supplemented with 10% heat‐inactivated FBS (GIBCO) and 1% Penicillin–Streptomycin. Cells were treated with Aβ_1–42_ oligomers at a strength of 2.5 μM from a stock of 200 μM for 24 h. Preparation of Amyloid Oligomers was done following the procedures reported earlier [18, 19].

### 2.2 Cell Transfection

All the transfections were done using Lipofectamine 2000 reagent (Invitrogen) following the manufacturer's protocol. For immunoprecipitation, cells were transfected with 2 μg of plasmids in a 60 mm format. For immunofluorescence and RNA‐FISH, cells were transfected with 500 ng of plasmids in a 12‐well format. Transfections were done for 6 h for both cell lines, followed by a media change. Plasmid‐transfected cells were then split to a larger growing surface after 24 h of transfection.

### 2.3 P‐Body Isolation

Confluent cells (~6 × 10^6^) cultured in a 90 mm dish were washed with cold 1X PBS (pH 7.4), harvested by scraping, and pelleted by centrifugation at 800× *g* for 5 min at 4°C. The cell pellet was resuspended in 500 μL of freshly prepared permeabilization buffer containing 50 ng/mL Digitonin in PBS and incubated for 10 min at 4°C with gentle rotation. Subsequent centrifugation at 2000× *g* for 2 min at 4°C yielded the soluble fraction in the supernatant and the non‐soluble components in the pellet. The pellet was washed with PBS and re‐centrifuged to remove residual Digitonin. For P‐body immunoisolation, 30 μL of Protein G agarose beads per sample were washed with IP buffer (20 mM Tris‐HCl pH 7.5, 150 mM KCl, 5 mM MgCl_2_, 1 mM DTT, 1X PMSF, and 20 U/mL RNase inhibitor), blocked with 5% BSA in lysis buffer for 1 h at 4°C, and incubated with anti‐Dcp1a antibody (1:100 dilution) in lysis buffer (20 mM Tris‐HCl pH 7.5, 150 mM KCl, 5 mM MgCl_2_, 1 mM DTT, 1X PMSF, and 20 U/mL RNase inhibitor and 0.5% Triton X‐100) for 4 h at 4°C with rotation. The non‐soluble pellet was lysed in 350 μL of lysis buffer. For C6 cells, the lysate was sonicated (3 cycles of 10 pulses each at 1 pulse per second) and centrifuged at 16 000× *g* for 10 min at 4°C. For PC12 cells, sonication was omitted, and the lysate was directly centrifuged at 3000× *g* for 10 min at 4°C. The cleared lysate was incubated overnight at 4°C with the antibody‐bound beads. The following day, the beads were washed three times with IP buffer and divided for downstream protein (western blot) and RNA (qRT‐PCR) analyses.

### 2.4 RNA Isolation and qRT‐PCR

Total RNA was isolated by using TRIzol or TRIzol LS reagent (Invitrogen) according to the manufacturer's protocol. mRNA levels were measured by a two‐step Real‐time qRT‐PCR method using Eurogentec Reverse Transcriptase Core Kit and MESA GREEN qPCR Master Mix Plus for SYBR Assay. A 100‐ to 200‐ng RNA was used for the detection of cellular mRNA levels, and an equal volume of RNA was used to analyze Dcp1a‐associated mRNA levels. GAPDH was used as an endogenous control. All the PCRs were done in the 7500 Applied Biosystem Real‐Time system. The Reverse Transcription reaction conditions were 25°C for 10 min, 48°C for 30 min, 95°C for 5 min, and finally, the product was held at 4°C. The qPCR conditions were 95°C for 5 min followed by 40 cycles of 95°C for 15 s, 60°C for 1 min. In our study, the mRNAs quantified following Dcp1a immunoprecipitation were normalized to the Dcp1a protein band intensity in each IP sample, measured to account for differences in the amount of Dcp1a isolated. The mRNA signals were then expressed relative to this normalized Dcp1a signal, ensuring accurate representation of PB‐associated transcript enrichment.

### 2.5 Western Blot

The samples were diluted in 5X sample loading buffer containing 312.5 mM Tris‐HCl pH 6.8, 10% SDS, 50% glycerol, 250 mM DTT, 0.5% Bromophenol blue and heated at 95°C for 10 min. Following SDS‐polyacrylamide gel electrophoresis, proteins were transferred to PVDF membranes (Millipore). Membranes were then blocked using 1X TBS (Tris‐buffered saline) supplemented with 0.1% Tween‐20 (1X TBST) and 3% BSA (HiMedia). Primary antibodies were added in 1X TBST containing 3% BSA for around 16 h at 4°C. After overnight incubation with antibody, the membranes were washed thrice for 5 min each with 1X TBST at room temperature to remove excess primary antibodies. It was then followed by incubation of membranes at room temperature for 1–1.5 h with secondary antibodies conjugated with horseradish peroxidase (1:8000 dilution) in 3% BSA containing 1X TBST. Excess secondary antibodies were washed thrice with 1X TBST at room temperature. Antigen–antibody complexes were detected with West Pico Chemiluminescent, Luminata Forte Western HRP substrate or West Femto Maximum Sensitivity substrates using the standard manufacturer's protocol. Imaging of all western blots was done using a UVP BioImager 600 system equipped with Vision Works Life Science software (UVP) V6.80.

### 2.6 Immunofluorescence

Cells grown on coverslips were fixed with 4% paraformaldehyde dissolved in 1X phosphate‐buffered saline (PBS) at room temperature for 30 min in the dark. Coverslips were then washed thrice with 1X PBS for 5 min each, which was followed by blocking and permeabilization with 1X PBS containing 10% goat serum (Gibco), 20% bovine serum albumin (HiMedia), and 0.1% Triton X‐100 (CALBIOCHEM) for 30 min at room temperature. After being washed thrice with 1X PBS for 5 min each, coverslips were then incubated with primary antibody, diluted in 1X PBS with 20% BSA and kept overnight in a humid chamber at 4°C. Following three washes with 1X PBS for 5 min each, secondary antibody labeled with Alexa Fluor diluted in 1X PBS containing 20% BSA was added on coverslips, and the incubation was done in a humid chamber for 1 h at room temperature in the dark. To remove excess secondary antibodies, the coverslips were washed thrice with 1X PBS for 5 min each and mounted with Vectashield mounting medium with DAPI (4′,6‐diamidino‐2‐phenylindole; Vector).

### 2.7 Cy3 Labelling of Oligos

Thirty microliters of DMSO containing one vial of CY3 monoreactive dye (GE HealthCare) was mixed with 10 μg of oligos against RL sequences re‐suspended in 0.1 M NaHCO_3_ buffer (pH 8.8) and kept for 24 h at room temperature in the dark. To remove unreacted dye, it was followed by two rounds of Ethanol precipitations and three rounds of purifications using miniQuick Spin Oligo Columns (Roche). The oligos were obtained from Eurogentec, having sequences as follows: RL 1: 5'aT*cacaaagatgatT*ttctttggaaggtT*ca; RL 2: 5'aT*tagctggaggcagcgT*taccatgcagaaa; and RL 3: 5'aT*agtccagcacgtT*catttgcttgcagT*ga.

### 2.8 RNA‐FISH (Fluorescence In Situ Hybridization)

Cells grown on coverslips were fixed with 4% paraformaldehyde dissolved in 1X PBS at room temperature for 30 min in the dark. Following three washes with 1X PBS for 5 min each, the coverslips were permeabilized with 70% alcohol overnight at 4°C. Cells were then rehydrated with 25% Formamide containing 2X SSC at room temperature for 10 min and incubated with a hybridization solution containing 10% Dextran Sulphate, 2 mM Vanadyl Ribonucleoside Complex, 0.02% RNase‐free BSA, 40 μg Salmon Sperm DNA, 2X SSC, 25% Formamide, 30 ng probe at 37°C overnight in a humid chamber. After being washed twice with 2X SSC containing 20% Formamide for 15 min each at 37°C, coverslips were mounted with Vectashield mounting medium with DAPI (4′,6‐diamidino‐2‐phenylindole; Vector).

### 2.9 Preparation of Amyloid Beta

One hundred percent of 1,1,1,3,3,3, hexafluoro‐2‐propanol (HFIP) was used to solubilize lyophilized Aβ_(1–42)_ (American Peptide) to a final stock concentration of 1 mM. HFIP was then removed by evaporation using SpeedVac (Eppendorf). The pellet was solubilized in anhydrous DMSO followed by sonication in a bath sonicator for 40 min at 37°C. The final stock solution was made at a concentration of 5 mM and stored at −80°C. For preparing oligomers of both Aβ_(1–42)_ and Aβ_(1–40)_, the stock peptides were diluted in 1X PBS and sodium dodecyl sulphate (SDS) at a concentration of 0.2% to make the concentration of the peptide 400 μM. The solution was then incubated overnight at 37°C in a rotating motion. It was then further diluted with 1X PBS to a final concentration of 200 μM and again incubated overnight at 37°C for proper oligomerization before use.

### 2.10 Image Capture and Post‐Capture Image Processing

All the images were captured using Zeiss LSM800 confocal microscope and Leica confocal microscope SP8 (Leica Microscope Systems; Wetzlar, Germany). All image processing was done using Imaris7 software developed by BITPLANE AG Scientific software.

### 2.11 Statistical Analysis

All graphs and statistical analyses were generated using Prism (v5.00 and v8.00) software (GraphPad, San Diego, CA). Nonparametric two‐tailed paired *t‐*tests were used for analysis. *p* values of < 0.05 were considered statistically significant, and *p* values of > 0.05 were not significant. Error bars indicate means ± standard deviation (SDs).

## 3 Results

### 3.1 Enrichment of RNA Processing Bodies in Detergent‐Permeabilized Cells Depleted of Cytosolic Content

**FIGURE 1:**
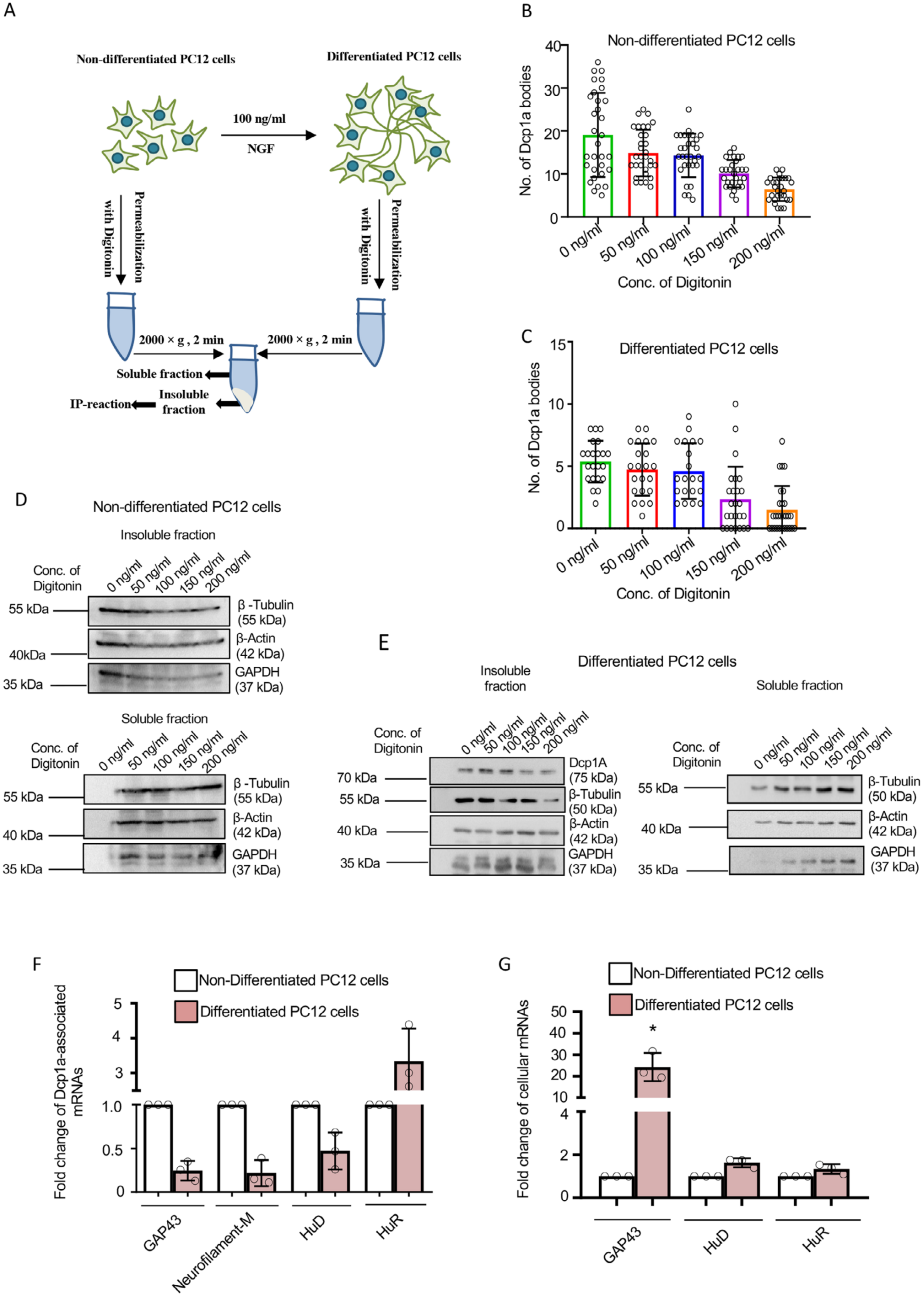
Detergent permeabilization of PC12 cells to enrich RNA processing bodies for downstream extraction and analysis. (A) Diagram showing separation of soluble and non‐soluble fractions from undifferentiated or 72‐h ‐NGF‐differentiated PC12 cells permeabilized with Digitonin. (B) Quantification of GFP‐Dcp1a bodies in undifferentiated PC12 cells permeabilized with increasing Digitonin concentrations (0, 50, 100, 150, 200 ng/mL) for 10 min at 4°C. (C) Quantification of GFP‐Dcp1a bodies in 72‐h differentiated PC12 cells (with 100 ng/mL NGF) permeabilized with increasing Digitonin concentrations (0–200 ng/mL) for 10 min at 4°C. (D) Western blots of endogenous Dcp1a, β‐Tubulin, β‐Actin, and GAPDH in non‐soluble (upper) and soluble (lower) fractions of undifferentiated PC12 cells permeabilized with Digitonin (0, 50, 100, 150, 200 ng/mL). Molecular weight markers are shown. (E) Western blot of endogenous Dcp1a, β‐Tubulin, β‐Actin, and GAPDH in non‐soluble (left) and soluble (right) fractions of 72‐h NGF‐differentiated PC12 cells permeabilized with increasing Digitonin (0–200 ng/mL). Molecular weight markers included. (F) qRT‐PCR quantified GAP43, Neurofilament‐M, HuD, and HuR mRNA levels normalized to relative pulled‐down Dcp1a levels. Endogenous Dcp1a was immunoprecipitated from undifferentiated or 72‐h NGF‐differentiated PC12 cells permeabilized with 50 ng/mL Digitonin [*p* = 0.0071 (GAP43), *p* = 0.0120 (Neurofilament‐M), *p* = 0.0495 (HuD), *p* = 0.0490 (HuR), *n* = 3]. (G) qRT‐PCR measured GAP43, HuD, and HuR mRNA levels normalized to GAPDH in total RNA from undifferentiated or 72‐h NGF‐differentiated PC12 cells [*p* = 0.0254 (GAP43), *p* = 0.0352 (HuD), *p* = 0.1195 (HuR), *n* = 3]. Data represents means ± SDs; ns, non‐significant, *p < 0.05, **p < 0.01. *p* values were obtained by using a two‐tailed paired Student's *t*‐test.

In this study, we aimed to develop a biochemical method using digitonin‐mediated selective plasma membrane permeabilization to remove cytosolic content via leaks created by the detergent, resulting in ghost cells that retain phase‐separated structures, including RNA PBs. From this, Dcp1a‐positive RNA PBs were isolated from both control and NGF‐differentiated PC12 cells. This approach enabled us to systematically examine changes in the association of target mRNAs with Dcp1a‐positive PBs during neuronal differentiation by removing signals from free cytosolic mRNAs (Figure 1A). The experiment involved selectively permeabilizing the cell membrane with digitonin, which released cytosolic content without significantly disrupting cellular structures or organelles. We previously used this method to produce ghost cells with intact P‐bodies, mitochondria, and ER structures while allowing the free passage of externally added molecules to study protein localization and PB association [22]. Increasing digitonin concentrations start to affect the integrity and number of PBs in both non‐differentiated and differentiated PC12 cells (Figure S1A,B and Figure 1B,C). After assessing β‐tubulin release levels in digitonin‐treated cells, we chose 50 ng/mL as the optimal condition because it maintains PB integrity while allowing cytosolic protein leakage, thereby keeping organellar structures and PBs free of cytosolic contamination in detergent‐treated cells. Western blot analyses showed a gradual decrease in soluble cytosolic proteins with higher detergent concentrations in the insoluble pellet (Figure 1D,E). Through optimization, we found that 50 ng/mL digitonin effectively permeabilizes both undifferentiated and differentiated PC12 cells, extracting a significant portion of soluble proteins while preserving the integrity of the non‐soluble, plasma membrane‐associated PBs, which should be enriched in PB‐specific proteins and mRNAs. This provides starting material for the immunoselection of PBs and associated RNA.

### 3.2 Isolation and Quantification of RNA Associated With RNA Processing Bodies From Naïve and Differentiated PC12 Cells

To explore the differential association of specific mRNAs encoding neuronal differentiation markers and RNA‐binding proteins (RBPs) with PBs, we immunoprecipitated the PB‐associated Dcp1a from detergent‐permeabilized cytosol‐free ghost cell extracts from both undifferentiated and differentiated PC12 cells. Dcp1a is a PB marker, and digitonin solubilization of cytoplasmic contents should enrich for PB‐localized mRNAs in immunoprecipitated samples obtained with Dcp1a‐specific antibodies (Figure 1A,F–G). We focused on four target mRNAs: two that encode important differentiation markers, GAP43 and Neurofilament‐M, which are expected to increase in expression in differentiated PC12 cells, and two that encode RBPs, specifically the neuron‐specific HuD and the ubiquitously expressed HuR. Our analysis of cellular and Dcp1a‐associated mRNAs indicated enrichment of HuR mRNAs associated with Dcp1a. In contrast, the association of GAP43, HuD, and Neurofilament‐M mRNAs with Dcp1a decreased, despite their increased expression in PC12 cells during differentiation. This supports the hypothesis that PB targeting of mRNA or its interaction with PB components, such as Dcp1a, and its cytosolic abundance are inversely related. Quantitative qRT‐PCR showed that during neuronal differentiation, mRNAs encoding differentiation markers such as GAP43 and Neurofilament‐M may have escaped PB targeting, reflecting possible derepression from miRNA‐mediated translation repression supporting PC12 differentiation [23]. Similarly, the association of HuD mRNA with PBs diminishes during differentiation, indicating greater availability for translation. In contrast, the mRNA for HuR, an RNA binding protein expressed across many cell types and involved in cell growth and proliferation, shows increased localization to PBs after differentiation, likely reflecting the need to downregulate HuR during neuronal differentiation (Figure 1F,G).

### 3.3 Aβ_1–42_ Oligomers Affect mRNA Association With RNA Processing Bodies in Differentiated PC12 Cells

**FIGURE 2:**
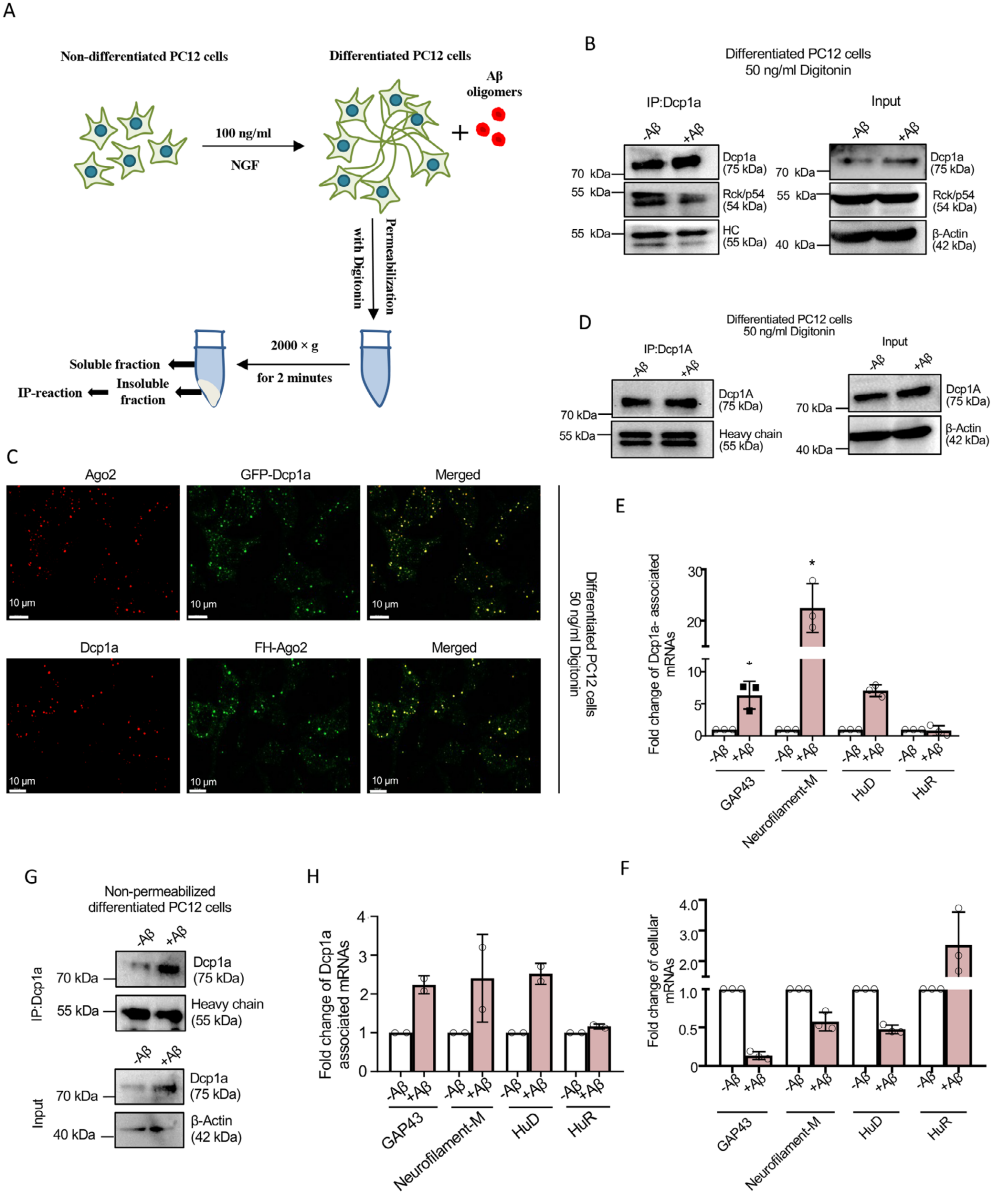
Altered association of mRNAs with PB‐associated Dcp1a in amyloid beta oligomer‐treated differentiated PC12 cells. (A) Schematic representation showing separation of soluble and non‐soluble fractions from Aβ_(1–42)_ ‐treated NGF‐differentiated PC12 cells permeabilized with Digitonin. (B) Immunoprecipitation of endogenous Dcp1a protein from 0 or 2.5 μM Aβ_(1–42)_ treated (24 h) differentiated PC12 cells permeabilized with 50 ng/mL Digitonin. Western blot images of pulled‐down Dcp1a and associated RCK/p54 levels in Dcp1a immunoprecipitated materials isolated from extract made with Digitonin‐permeabilized and PB‐enriched ghost NGF‐differentiated PC12 cells. Dcp1a and RCK/p54 levels, along with β‐Actin as a loading control, are shown in the inputs used for immunoprecipitation. Molecular weight markers are indicated. HC, heavy chain. (C) Confocal Images showing co‐localization between exogenously expressed GFP‐Dcp1a (Green) and endogenous Ago2 (Red) in 72‐h differentiated (with 100 ng/mL NGF) PC12 cells permeabilized with 50 ng/mL Digitonin (upper panel). Confocal Images showing co‐localization between exogenously expressed FH‐Ago2 (Green) and endogenous Dcp1a (Red) in 72‐h differentiated PC12 cells permeabilized with Digitonin (lower panel). Merged images are shown. Fields have been detected in 63× magnification. Scale bar represents 10 μm. (D, E) Immunoprecipitation of endogenous Dcp1a protein from 0 or 2.5 μM Aβ_(1–42)_ treated (24 h) differentiated PC12 cells permeabilized with 50 ng/mL Digitonin. Western blot images of the pulled‐down Dcp1a levels and input Dcp1a levels, with β‐Actin as a loading control, are shown. Molecular weight markers are indicated. HC, heavy chain (D). qRT‐PCR based quantification of pulled‐down Dcp1a associated GAP43, Neurofilament‐M, HuD and HuR mRNA levels normalized against relative immunoprecipitated Dcp1a levels [*p* = 0.0496 (for GAP43), *p* = 0.0160 (for Neurofilament‐M), *p* = 0.0076 (for HuD), *p* = 0.7230 (for HuR), *n* = 3 independent experiments] (E). (F) qRT‐PCR based quantification of cellular GAP43, Neurofilament‐M, HuD and HuR mRNA levels normalized against GAPDH level in 0 or 2.5 μM Aβ_(1–42)_ treated (24 h) differentiated PC12 cells permeabilized with 50 ng/mL Digitonin [*p* = 0.0011 (for GAP43), *p* = 0.0260 (for Neurofilament‐M), *p* = 0.0038 (for HuD), *p* = 0.1306 (for HuR), *n* = 3 independent experiments]. Molecular weight markers are shown. Data represents means ± SDs; ns, non‐significant, *p < 0.05, **p < 0.01. *p* values were obtained by using two‐tailed paired Student's *t* test. (G, H) Immunoprecipitation of total endogenous Dcp1a protein from both 0 or 2.5 μM Aβ_(1–42)_ treated differentiated PC12 cells without digitonin permeabilization. Western blot images of the pulled‐down Dcp1a level and input Dcp1a levels, with β‐Actin as a loading control, are shown. Molecular weight markers are indicated. HC, heavy chain (G). qRT‐PCR‐based quantification of associated GAP43, Neurofilament‐M, HuD, and HuR mRNA levels normalized against relative levels of Dcp1a immunoprecipitated (*n* = 2 independent experiments) (H).

To investigate how amyloid proteins interfere with the neuronal differentiation process, we treated differentiated PC12 cells with 2.5 μM amyloid beta Aβ_(1–42)_ and then immunoprecipitated Dcp1a from digitonin‐permeabilized cells (Figure 2A). Differentiated PC12 cells treated with digitonin also retain Ago2 and RCK/p54 in the Dcp1a‐positive bodies used for Dcp1a IP and RNA extraction (Figure 2B,C). Dcp1a was found to be associated with RCK/p64, a marker protein of RNA PBs, in immunoprecipitation (Figure 2B). RNA analysis revealed a significant increase in the association of GAP43, Neurofilament‐M, and HuD mRNAs—transcripts that were found to be dissociated from PBs during NGF‐mediated PC12 differentiation (Figure 1F,G)—with PB‐associated Dcp1a isolated following Aβ_1–42_ oligomers treatment (Figure 2D,E). These results suggest that Aβ_1–42_ oligomers disrupt mRNA escape from PBs in differentiating PC12 cells and possibly promote the translational repression of differentiation‐related protein‐coding mRNAs, such as Neurofilament‐M, GAP43, and HuD, by inducing their PB‐targeting in differentiated PC12 cells treated with Aβ_1–42_ oligomers.

As a result, levels of these mRNAs in cells decreased after exposure to Aβ_1−42_ oligomers, which contrasts with the upregulation seen during NGF‐induced differentiation (Figure 2F). This decrease suggests that Aβ_1–42_ oligomers may hinder the proper translation of these essential mRNAs by promoting their targeting to PBs and subsequent downregulation, thereby disrupting the differentiation process. Interestingly, an increase in Dcp1a association with GAP‐43, Neurofilament‐M, and HuD mRNAs was also observed when Dcp1a immunoprecipitation was carried out from total cell lysates without prior digitonin permeabilization. However, the relative enrichment of GAP‐43, HuD, and Neurofilament‐M mRNAs with total cellular Dcp1a was much less compared to mRNAs associated with Dcp1a isolated from PBs (Figure 2G,H). Thus, PB enrichment was found to help reduce noise and enhance signals of PB‐localized mRNAs, using PB‐specific proteins such as Dcp1a immunoprecipitated from the PB‐enriched fraction, thereby enabling the identification and quantification of mRNAs altered specifically in PBs by Aβ_1−42_ oligomers in NGF‐differentiated PC12 cells.

### 3.4 Aβ_1–42_ Oligomers Affect mRNA Association With RNA Processing Bodies in Glioblastoma Cells

**FIGURE 3:**
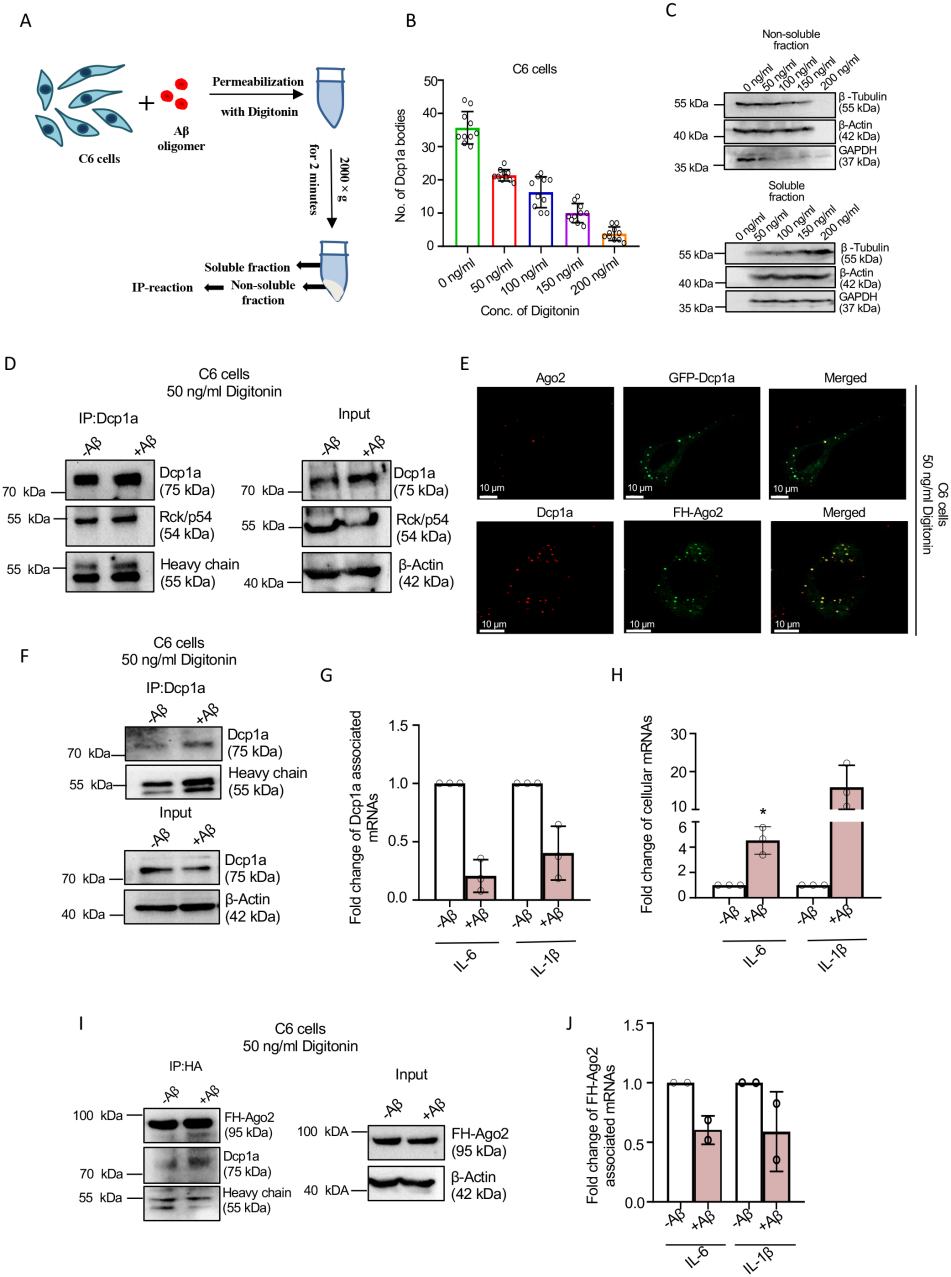
Changed pattern of mRNA association with PB‐localized Dcp1a in amyloid beta‐treated astroglial cells. (A) Schematic representation showing separation of soluble and non‐soluble fractions from amyloid beta oligomer‐treated C6 cells permeabilized with Digitonin. (B) Quantification of GFP‐Dcp1a bodies in GFP‐Dcp1a expressing C6 cells permeabilized with increasing concentration of Digitonin (0, 50, 100, 150, 200 ng/mL) for 10 min at 4°C. (C) Western blot of endogenous β‐Tubulin, β‐Actin and GAPDH were done to measure the expression levels of these proteins in the non‐soluble fraction (upper panel) and soluble fraction (lower panel) of C6 cells permeabilized with increasing concentration of Digitonin (0, 50, 100, 150, 200 ng/mL). Molecular weight markers are shown. (D) Immunoprecipitation of endogenous Dcp1a protein from both 0 or 2.5 μM Aβ_(1–42)_ treated (24 h) C6 cells permeabilized with 50 ng/mL Digitonin. Western blot images of immunoprecipitated Dcp1a and associated RCK/p54 levels, and input Dcp1a and RCK/p54 levels with β‐Actin as loading control are shown. Molecular weight markers are also shown. HC, heavy chain. (E) Confocal Images showing co‐localization between exogenously expressed GFP‐Dcp1a (Green) and endogenous Ago2 (Red) in C6 cells permeabilized with 50 ng/mL Digitonin (Upper panel). Confocal Images showing co‐localization between exogenously expressed FH‐Ago2 (Green) and endogenous Dcp1a (Red) in C6 cells permeabilized with 50 ng/mL Digitonin (lower panel). Merged images are shown. Fields have been detected in 63× magnification. Scale bar represents 10 μm. (F, G) Immunoprecipitation of endogenous Dcp1a protein from both 0 or 2.5 μM Aβ_(1–42)_ treated C6 cells permeabilized with 50 ng/mL Digitonin. Western blot images of immunoprecipitated Dcp1a level, and input Dcp1a level with β‐Actin as loading control, are shown. Molecular weight markers are also shown. HC, heavy chain (F). qRT‐PCR‐based quantification of immunoprecipitated Dcp1a‐associated IL‐6 and IL‐1β mRNA levels normalized against relative immunoprecipitated Dcp1a levels [(*p* = 0.0102 (for IL‐6), *p* = 0.0463 (for IL‐1β), *n* = 3 independent experiments)] (G). (H) qRT‐PCR‐based quantification of cellular IL‐6 and IL‐1β mRNA levels normalized against GAPDH level in 0 or 2.5 μM Aβ_(1–42)_ treated C6 cells permeabilized with 50 ng/mL Digitonin [(*p* = 0.0301 (for IL‐6), *p* = 0.0472 (for IL‐1β), *n* = 3 independent experiments)]. Data represents (panels G and H) means ± SDs; ns, non‐significant, *p < 0.05. *p* values were obtained by using a two‐tailed paired Student's *t*‐test. (I, J) Immunoprecipitation of exogenously expressed FH‐Ago2 protein by anti‐HA antibody from both 0 or 2.5 μM Aβ_(1–42)_ treated C6 cells permeabilized with 50 ng/mL Digitonin. Western blot images of immunoprecipitated FH‐Ago2 and associated Dcp1a levels, and input FH‐Ago2 level with β‐Actin as loading control are shown. Molecular weight markers are shown. HC, heavy chain (I). qRT‐PCR based quantification of HA‐Ago2 associated IL‐6 and IL‐1β mRNA levels normalized against relative pulled down FH‐Ago2 levels (*n* = 2 independent experiments) (J).

In glial cells treated with Aβ_1–42_ oligomers, elevated mRNA levels of pro‐inflammatory cytokines are reported to be linked to disrupted repressive microRNA (miRNA) function caused by retention of miRNPs in PBs and endosomes, resulting in loss of miRNP‐mRNA interaction [18, 19]. As miRNP interactions are considered a primary mechanism for delivering mRNAs to PBs, we expect a loss of cytokine mRNAs in PBs due to impaired interaction with repressive miRNPs [18]. To investigate the suggested loss of association of these inflammatory cytokine mRNAs with PBs upon exposure of glioblastoma cells to amyloidogenic Aβ_1–42_ oligomers, we used the detergent‐based permeabilization protocol to isolate phase‐separated, non‐soluble PBs‐associated Dcp1a from C6 astroglial cells (Figure 3A). After optimizing digitonin concentration for this assay in C6 cells, we confirmed that 50 ng/mL digitonin efficiently isolated PB‐associated Dcp1a bodies, consistent with prior optimization results with PC12 cells (Figure 3B,C, Figure S2A).

We immunoprecipitated Dcp1a from the non‐soluble fractions of digitonin‐permeabilized C6 cells, both untreated and Aβ_1–42_ oligomer treated, followed by qRT‐PCR quantification of PB‐associated pro‐inflammatory cytokine mRNAs IL‐6 and IL‐1β. The immunoprecipitated Dcp1a was found to be associated with RCK/p64, a PB marker, and confocal imaging revealed that Ago2 was also located with Dcp1a after digitonin‐permeabilization of C6 cells (Figure 3D,E). Our laboratory previously reported that 2.5 μM Aβ_1–42_ oligomer treatment for 24 h increases IL‐1β and IL‐6 expression at both mRNA and protein levels by 2–4‐fold in C6 cells [19]. However, we found that their association with PBs was significantly reduced, consistent with the enhanced total cellular mRNA levels observed (as PB components‐mediated decapping, deadenylation, and degradation are expected to happen for PB‐targeted messages) in the presence of amyloid oligomers [19] (Figure 3F–H). A similar decrease in total cellular Dcp1a association was observed when total Dcp1a was immunoprecipitated from non‐permeabilized C6 cells (Figure S2B,C), suggesting the escape of inflammatory cytokines from PBs in Aβ_1–42_ oligomer‐treated C6 cells. Ago2 is found to colocalize with PBs in control and Aβ‐treated cells, and we analyzed the interaction between FH‐Ago2 and cytokine mRNAs in detergent‐permeabilized FH‐Ago2‐expressing C6 cells. Immunoprecipitation assays revealed a significant reduction in the association of IL‐6 and IL‐1β mRNA with Ago2 following Aβ treatment, indicating that Aβ_1–42_ oligomers disrupt Ago2‐mRNA interactions, which may contribute to the increased polysome association and increased cytokine expression observed earlier [19], along with a loss of PB/Dcp1a association of those mRNAs (Figure 3H,J). Additionally, FH‐Ago2 proteins co‐immunoprecipitated with Dcp1a (Figure 3I) and co‐localized with Dcp1a in immunofluorescence assays (Figure 3E), supporting the interplay between these PB components in cytokine mRNA regulation and confirming the effectiveness of this technique to detect differential PB‐localization of mRNAs tested with multiple PB components (i.e., Dcp1a and Ago2).

### 3.5 Rheb‐Myc Rescue Cytokine mRNA Targeting to Processing Bodies in Aβ_1–42_ Oligomers‐Treated C6 Cells

**FIGURE 4:**
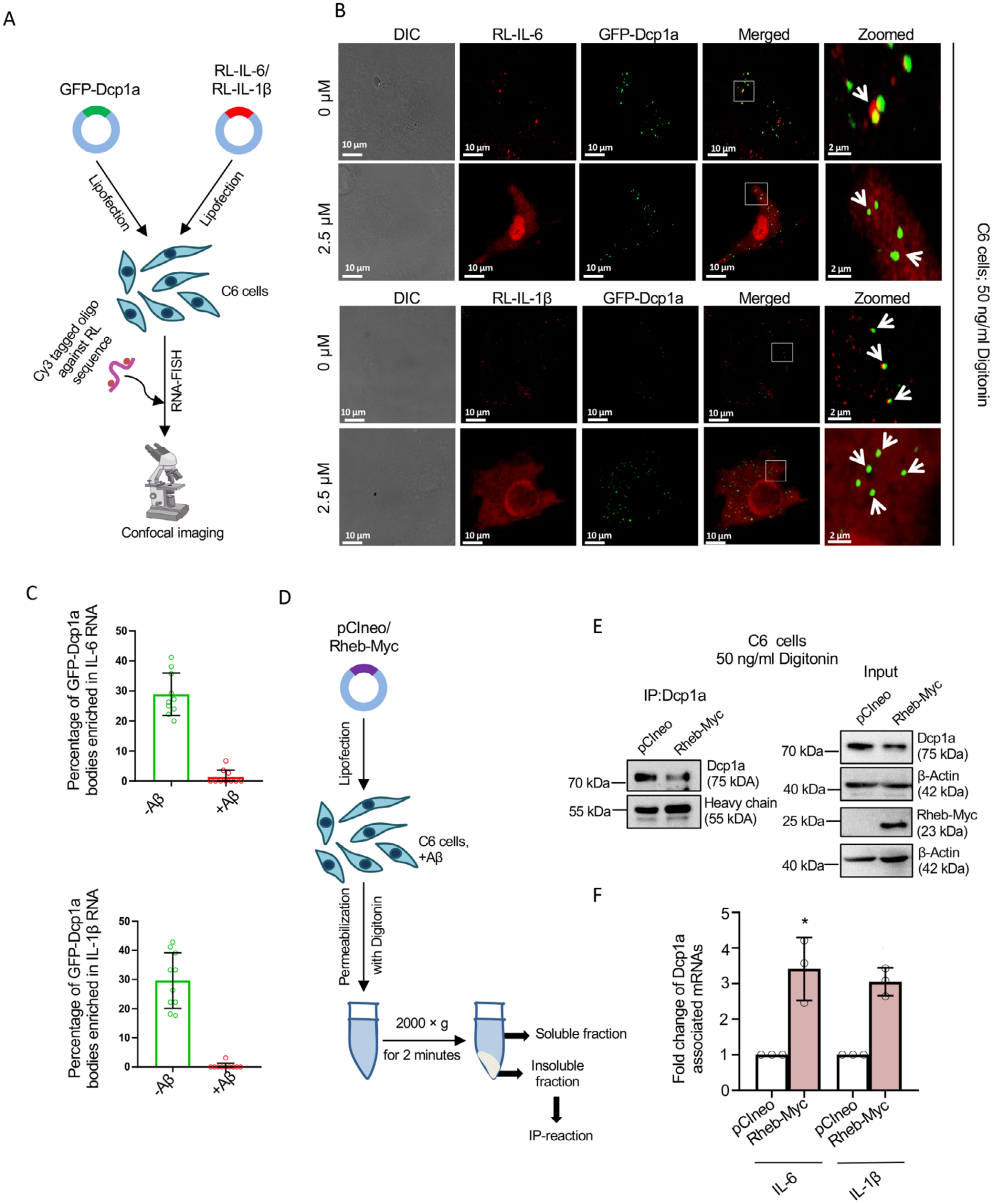
Rescue in Dcp1a‐association and PB‐localization of cytokine mRNAs by Rheb‐Myc in amyloid‐beta treated C6 cells. (A) Schematic representation of RNA‐FISH experiment using Cy3‐tagged oligos against RL sequence in GFP‐Dcp1a, and RL‐IL‐6, or RL‐IL‐1β co‐transfected amyloid beta oligomer‐treated C6 cells. (B) Confocal Images showing co‐localization between exogenously expressed GFP‐Dcp1a (Green) and RL‐IL‐6 mRNAs (Red) by RNA‐FISH in 0 or 2.5 μM Aβ_(1–42)_ treated C6 cells (Upper panel). Confocal Images showing co‐localization between exogenously expressed GFP‐Dcp1a (Green) and RL‐IL‐1β mRNAs (Red) by RNA‐FISH in 0 or 2.5 μM Aβ_(1–42)_ treated C6 cells (lower panel). Merged images are shown. Fields have been detected in 63× magnification. Scale bar of non‐zoomed images represents 10 μm, whereas those of zoomed images represent 2 μm. Arrowheads marking the GF‐PDcp1a bodies. (C) Quantification of the percentage of GFP‐Dcp1a, as in experiments described in panel (B), positive for RL‐reporters (*n* = 10). (D) Schematic representation showing separation of soluble and non‐soluble fractions from Aβ_(1–42)_ treated pCIneo or Rheb‐myc expressing C6 cells permeabilized with Digitonin. (E, F) Immunoprecipitation of endogenous Dcp1a protein from both pCIneo or Rheb‐Myc expressing 2.5 μM Aβ_(1–42)_ treated C6 cells permeabilized with 50 ng/mL Digitonin. Western blot images of immunoprecipitated Dcp1a levels and input Dcp1a and Rheb‐Myc levels, with β‐Actin as a loading control, are shown. Molecular weight markers are also shown. HC, heavy chain (E). qRT‐PCR based quantification of associated IL‐6 and IL‐1β mRNA levels normalized against relative pulled down Dcp1a levels [*p* = 0.0421 (for IL‐6), *p* = 0.0119 (for IL‐1β), *n* = 3 independent experiments] (F). Data represents means ± SDs; ns, non‐significant, *p < 0.05, ****p < 0.0001. *p* values were obtained by using a two‐tailed paired Student's *t*‐test.

In parallel, to confirm the changes in the PB association of specific pro‐inflammatory cytokine mRNAs, we used RNA fluorescence in situ hybridization (RNA‐FISH) techniques to verify our biochemical data [13]. C6 cells were co‐transfected with plasmids encoding GFP‐tagged Dcp1a and reporter constructs expressing Renilla luciferase (RL) fused to either IL‐6 or IL‐1β mRNA sequences (RL‐IL‐6 and RL‐IL‐1β). After permeabilization with 50 ng/mL digitonin, we applied Cy3‐labeled oligonucleotide probes complementary to the RL sequence to specifically detect these reporter mRNAs in C6 cells (Figure 4A).

Image analysis showed a significant increase in RL‐IL‐6 and RL‐IL‐1β mRNA signal intensity in cells treated with Aβ_1–42_ oligomers compared with untreated controls, indicating increased expression or stabilization of these cytokine transcripts. Notably, despite this rise in mRNA levels, there was a decrease in the co‐localization of RL‐tagged cytokine mRNAs with GFP‐Dcp1a‐positive PBs. We also did not observe any marked changes in cell morphology on expression of RL‐reporters or due to enhanced RL mRNAs in Aβ_1–42_ oligomer‐treated conditions. In situ data suggest that Aβ oligomers interfere with the targeting of cytokine mRNAs to PBs, possibly ensuring their availability for protein translation (Figure 4B,C). These RNA‐FISH results support our immunoprecipitation data, collectively indicating that amyloidogenic oligomers disrupt the regulation of cytokine mRNAs by PBs required for checked expression of cytokines in naïve glial cells, and contribute to their elevated expression in Aβ_1–42_ oligomer‐treated C6 cells. This dysregulation may partly drive the inflammatory response observed in C6 glioblastoma, primary astroglia, or rat brain exposed to Aβ_1–42_ oligomers [19], establishing a mechanistic link between amyloid pathology and the post‐transcriptional regulation of inflammatory mediators in Alzheimer's Disease context.

Interestingly, Rheb‐Myc, the activator of mTORC1, has been reported to rescue cytokine repression in Aβ_1–42_‐treated cells and to cause a marked decrease (about twofold) in cellular IL‐6 and IL‐1β levels [19]. In the Dcp1a‐IP reaction performed with Rheb‐Myc‐expressing cells treated with Aβ_1–42_ oligomers, we observed a rescue of Dcp1a association with IL‐1β and IL‐6, confirming the reciprocal relationship between the PB‐localization of cytokine mRNAs and the expression of Rheb‐Myc proteins. This rescue, mediated by Rheb‐Myc, the activator of mTORC1, underscores how amyloid treatment affects cytokine mRNA regulation (Figure 4D–F).

## 4 Discussion

Our findings expand the current understanding of how RNA processing bodies (P‐bodies or PBs) contribute to post‐transcriptional gene regulation. These specialized, dynamic cytoplasmic granules [11, 24, 25] appear to act as adaptable platforms whose composition and function vary depending on the cellular context. The ability of proteins and RNA molecules to undergo liquid–liquid phase separation provides the structural basis for PB formation [26], but our observations suggest that such assemblies are not static. Instead, the interactions that drive phase separation are likely modulated by cellular cues associated with mRNA decay, translational repression, and RNA surveillance [22]. This dynamic nature enables PBs to fine‐tune the balance between mRNA degradation and storage of translationally repressed transcripts [11, 24, 25].

By sequestering mRNAs in a translationally inactive state, PBs may regulate the temporal and spatial dynamics of protein synthesis, ensuring that translation occurs only when and where it is required. Such regulation becomes particularly important when cells experience stress, nutrient limitation, or developmental transitions, as PB‐mediated control can facilitate rapid adjustments in protein output [5, 14]. Our data support this model by showing that PB behavior is sensitive to environmental fluctuations and likely participates in coordinating the adaptive gene expression response, including in response to amyloid proteins.

In neurons, this level of translational control is especially critical. Neuronal morphology and function rely on precise regulation of mRNA localization and translation, both in perinuclear regions and at distant synapses [5, 15]. PBs, through their capacity to modulate mRNA availability, may thus influence key neuronal processes such as synaptic plasticity, learning, and memory. Disruption of PB dynamics or loss of translational control has been associated with several neurodegenerative conditions, including Alzheimer's, Parkinson's, and Huntington's diseases [27]. Therefore, examining how PBs reorganize and regulate mRNA fate under different physiological or stress conditions can provide valuable insights into the molecular mechanisms that underlie these disorders.

A significant advancement in PB research was made by Hubstenberger et al. in 2017, who utilized Fluorescence‐Activated Particle Sorting (FAPS) to purify PBs from human epithelial cells. This high‐throughput technique allowed for the identification of hundreds of proteins and RNA molecules that comprise PBs, providing a detailed view of these RNA‐protein granules [28]. However, FAPS has limitations, including the need for specialized equipment such as high‐throughput fluorescence sorters and the potential to introduce artifacts during sorting and purification. These constraints can hinder the complete characterization of PBs under various experimental conditions, particularly in settings with limited access to advanced instruments. A recent report by Sun et al. introduced a modified FACS‐based method for isolating PBs. This approach leverages PB‐TRIBE‐STAMP, a tool based on two orthogonal RNA editing enzymes that require high expression of fusion proteins and ADAR2‐LSm14‐driven RNA editing as criteria to identify PB‐associated RNAs. However, off‐target RNA editing remains a significant concern, potentially affecting the certainty that the identified mRNAs are genuinely associated with PBs. Additionally, these conditions might not fully replicate the physiological context, which is essential for accurate PB and mRNA analysis. Despite these limitations, the method offers a promising avenue for high‐throughput RNA transcript analysis [29]. A comparative study of these methods is shown in Table 1.

**TABLE 1:** Comparative advantages and limitations of RNA processing body (PB) isolation and RNA analysis using different strategies.

Article	Methods used	mRNAs detected	Limitations	Physiological relevance
Hubstenberger et al., *Molecular Cell*, 2017	Fluorescence‐Activated Particle Sorting (FAPS) of GFP‐LSM14A‐labeled PBs followed by RNA‐seq and proteomics	Thousands of translationally repressed mRNAs enriched for regulatory genes—e.g., *DDX6*, *4E‐T*, *PUM1*, *IGF2BP1*‐associated transcripts	Requires fluorescent tagging and specialized flow sorter; may miss low‐abundance mRNAs; mechanical stress may perturb PB integrity	Demonstrated PBs as condensation hubs for translationally repressed regulons rather than decay sites; revealed compositional complexity of human PBs
Safieddine et al., *Molecular Cell*, 2024	FAPS‐based PB purification synchronized across cell‐cycle stages + single‐molecule FISH validation	~4000–5000 PB‐enriched mRNAs; examples: *TOP2A*, *CLK1*, *FBXO5*, *CCNE2*	Large cell numbers required; smFISH limited to selected transcripts; quantitative variation across cycle phases	PB transcriptome dynamically changes through cell cycle; PBs sequester cell‐cycle‐regulated mRNAs when encoded proteins are dispensable
Sun et al., *Nature Communications*, 2025	PB‐TRIBE‐STAMP (APOBEC1‐DDX6 + LSM14A‐ADAR2dd fusion) coupled with fluorescent PB isolation and long‐read RNA‐seq	1639 (HCT116) and 2577 (HEK293T) PB‐associated mRNAs; examples: *XBP1*, *SREBF2*, *HMGA2*	Possible off‐target RNA editing; requires large‐scale expression of fusion proteins; computationally intensive	PB association correlates with shortened poly(A) tails and isoform‐specific sequestration; PB‐mRNA association dynamically regulated during UPR and cell‐cycle transitions
Current manuscript by Ray et al. 2025	Digitonin‐based selective permeabilization to deplete cytosolic content, enrich phase‐separated PBs; Dcp1a‐immunoprecipitation from detergent‐insoluble fraction; mRNAs quantified by qRT‐PCR and validated by RNA‐FISH	Neuronal PBs: *GAP43*, *Neurofilament‐M*, *HuD*, *HuR*; Astroglial PBs: *IL‐6*, *IL‐1β*	Captures Dcp1a‐positive PB subset only; may not reflect full PB heterogeneity; semi‐quantitative vs. RNA‐seq; dependent on antibody efficiency	Amyloid‐β promotes PB sequestration of neuronal differentiation mRNAs and escape of cytokine mRNAs in glia; Rheb‐Myc restores cytokine mRNA PB‐localization, highlighting a therapeutic axis for neuroinflammation and PB‐mediated RNA control

To overcome these challenges, we developed a more accessible and flexible method for isolating PBs. Our approach employs a detergent‐based technique to separate the soluble and non‐soluble fractions of the cytoplasm, followed by solubilization, and removal of insoluble membranes by centrifugation to enrich and isolate PBs. We selected digitonin as the detergent due to its ability to selectively permeabilize the plasma membrane in a controlled manner, thereby maintaining cellular integrity and preserving PB structure [30]. The non‐soluble, phase‐separated fraction, which contains PBs, is then enriched by centrifugation. To further isolate PBs, we performed immunoisolation using an antibody against Dcp1a, a highly abundant marker protein of PBs. This step enables the selective capture of PBs and their associated mRNAs.

Our analysis of mRNA content in isolated PBs using quantitative reverse transcription polymerase chain reaction (qRT‐PCR) provides new insights into the selective recruitment of transcripts into these granules across different cellular contexts. Rather than serving as passive repositories, PBs appear to dynamically associate with specific mRNAs in response to the cell's physiological state. PB's role in neuronal differentiation and survival has been noted before [5, 23]. The enrichment patterns we observed for mRNAs linked to neuronal differentiation suggest that PBs may fine‐tune the availability of transcripts required for neuronal development and function. Such selective association points toward an active role of PBs in coordinating the temporal regulation of differentiation‐associated mRNAs, rather than merely reflecting general translational repression.

In the context of amyloidogenic stress, our findings further reveal that proteins such as amyloid‐beta can profoundly influence PB‐associated mRNA populations. Given that amyloid aggregation is a hallmark of neurodegenerative disorders, the altered mRNA localization we observed likely represents one mechanism by which these proteins perturb post‐transcriptional regulation [18, 19]. Disruption of PB integrity or function under these conditions may compromise mRNA surveillance and translational control, thereby contributing to neuronal vulnerability [22]. Furthermore, our observations in glial cells indicate that amyloid‐induced inflammation affects PB dynamics, leading to reduced sequestration of pro‐inflammatory cytokine mRNAs. This highlights a broader, cell‐type–specific role of PBs in modulating RNA fate during neuroinflammatory responses.

The identification of Dcp1a‐positive granules as bona fide PBs was supported by their co‐localization with additional PB markers such as Ago2 and Rck/p54, strengthening the validity of our findings across both neuronal and glial systems. Collectively, these results underscore the plasticity of PB composition and function, suggesting that PBs act as dynamic modulators of RNA metabolism whose behavior is closely linked to cellular differentiation, stress, and inflammatory signaling. Post‐transcriptional modifications, such as mRNA degradation, translation, and sequestration in PBs, are essential for maintaining cellular homeostasis. Disruptions in these processes can lead to a wide range of diseases, including neurological disorders, cancer, and inflammatory conditions. Our study demonstrates that the method we developed for isolating and characterizing PBs can be widely applied to investigate mRNA association with PBs in various cellular and physiological contexts. This approach holds significant potential for uncovering how PBs contribute to disease pathogenesis, opening new avenues for therapeutic strategies. Moving forward, we anticipate that this accessible and adaptable method will become a valuable tool for studying the molecular mechanisms and importance of phase separation as a key post‐transcriptional regulation happening in health and disease.

These findings also hold important in vivo and clinical implications. Given that PBs regulate mRNA stability and translation, their dysregulation could contribute to transcriptomic imbalances observed in neurodegenerative diseases such as Alzheimer's, Parkinson's, and Huntington's. The observed disruption of PB‐associated mRNA localization under amyloidogenic stress suggests that PB dysfunction may represent an early molecular event preceding neuronal degeneration. Similarly, reduced sequestration of pro‐inflammatory mRNAs in glial PBs highlights a potential role for these structures in modulating neuroinflammatory responses. Thus, strategies aimed at preserving PB integrity or modulating phase separation dynamics may offer new therapeutic opportunities to restore mRNA homeostasis in neurodegenerative and inflammatory disorders. Extending this work to in vivo models could help validate PBs as both biomarkers and regulatory targets in disease progression.

While our detergent‐based method for isolating PBs offers a practical and accessible alternative to high‐throughput approaches like FAPS, it is not without limitations. PBs are inherently heterogeneous in their composition, and distinct subpopulations likely exist within a single cell, each potentially involved in different aspects of mRNA regulation [31]. Our assay, which relies on immunoprecipitation using the PB marker such as Dcp1a, captures only a representative subset of these structures. As such, the isolated PBs may not fully reflect the diversity of PB populations or their complete RNA content. Additionally, while our co‐localization with known PB markers supports the authenticity of these granules as “traditional” PBs, functional heterogeneity and context‐specific roles of PBs may still be underrepresented. Future refinements, including multi‐marker approaches and single‐particle analyses, may help uncover the full complexity of PB biology.

## Author Contributions

Sritama Ray: data curation, formal analysis, investigation, methodology. Kamalika Mukherjee: data curation, formal analysis, investigation, writing‐review and editing, conceptualization, methodology, supervision. Suvendra N. Bhattacharyya: data curation, formal analysis, investigation, writing – review and editing, conceptualization, funding acquisition, methodology, supervision.

## Funding

This work was supported by the UNMC|College of Public Health, University of Nebraska Medical Center and the UNMC StartUp Fund Bhattacharyya|College of Medicine, University of Nebraska Medical Center (COM, UNMC)‐Lieberman Research Fund 2025_Mukherjee, the Department of Science and Technology, Govt. of India‐SwarnajayantiFellowship (DST/SJF/LSA‐03/2014‐15). SERB, Department of Science and Technology, Govt. of India High‐RiskHigh‐Reward Grant (HRR/2016/000093) and CEFIPRA 9 (6003‐J).

## Disclosure

Supporting information: Information on plasmids, oligos, antibodies, and primers is available in Tables S1–S8.

Resource availability: All resources reported in this manuscript are available in the data presented within the manuscript figures and resource tables.

Lead contact: Further information and requests for resources and reagents should be directed to and will be fulfilled by the lead contact, Dr. Suvendra N. Bhattacharyya (sbhattacharyya@unmc.edu).

Materials availability: This study did not produce any unique reagents.

## Conflicts of Interest

The authors declare no conflicts of interest.

## Supporting information


**Data S1:** fsb271351‐sup‐0001‐Supinfo.pdf.

## Data Availability

All data supporting the findings of this study are available from the corresponding author. This paper does not report the original code. Any additional information required to reanalyze the data reported in this paper is available from the lead contact upon request.
