# Supplementary material for: Ras Homolog Enriched in Brain Protein Reverses Amyloid Beta‐Induced Escape of Inflammatory Cytokine mRNAs From Immunoisolated RNA Processing Bodies of Glioblastoma Cells
Source: FASEB J. 2025 Dec 13;39(24):e71351. doi: 10.1096/fj.202502872R (PMC12701684; doi:10.1096/fj.202502872R)
Supplement: Supplementary file 1 — Data S1: fsb271351‐sup‐0001‐Supinfo.pdf. [file FSB2-39-e71351-s001.pdf]

Supplementary Information

Ras Homolog Enriched in Brain Protein Reverses Amyloid Beta-Induced Escape of Inflammatory Cytokine mRNAs from Immunoisolated RNA Processing Bodies of Glioblastoma Cells

Sritama Ray[1], Kamalika Mukherjee[2, #], and Suvendra N. Bhattacharyya[3, #]

[1]CSIR-Indian Institute of Chemical Biology, Kolkata, West Bengal 700032, India

[2]Departments of Anesthesiology, University of Nebraska Medical Center, Omaha, NE 68198-5880

[3]Department of Pharmacology and Experimental Neuroscience, University of Nebraska Medical Center, Omaha, NE 68198-5880

Supplementary figures and legends

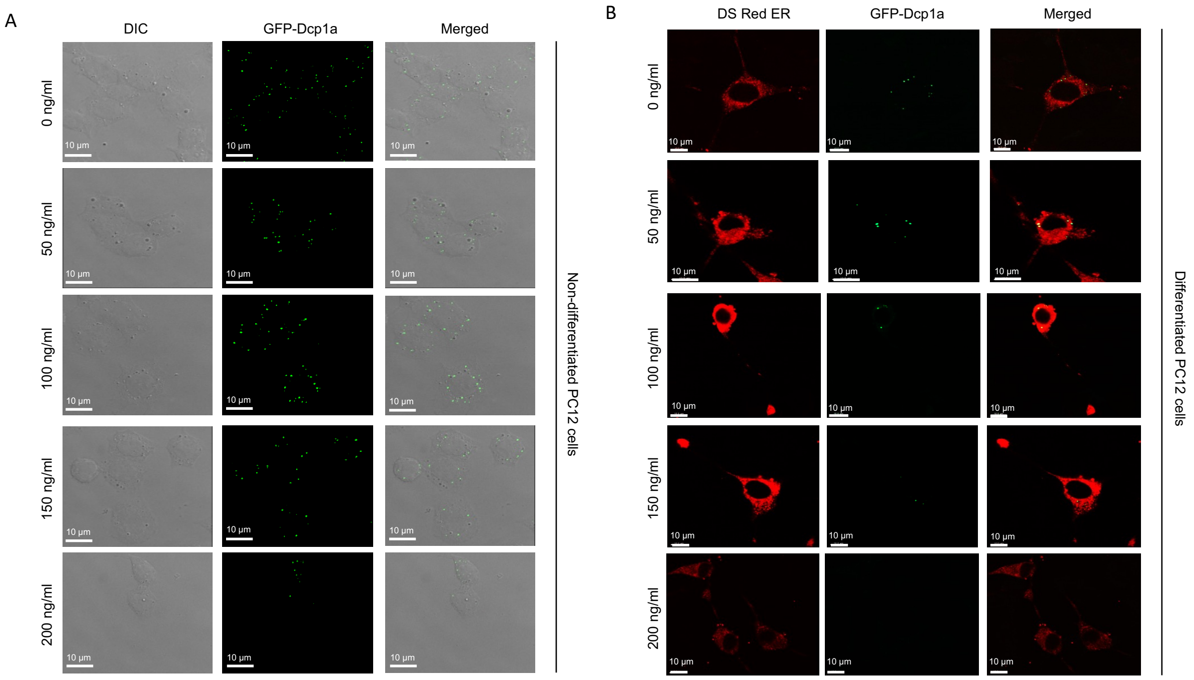

Figure S1 Digitonin permeabilization effects on internal P-bodies in PC12 cells. Related to Figure 1.

(A) Confocal Images of GFP-Dcp1a (Green) expressing non-differentiated PC12 cells permeabilized with increasing concentration of Digitonin (0, 50, 100, 150, 200 ng/ml) for 10 minutes at 4 °C. Merged images are shown.

(B) Confocal Images of DS Red ER (Red) and GFP-Dcp1a (Green) expressing 72 hours differentiated (with 100 ng/ml NGF) PC12 cells permeabilized with increasing concentration of Digitonin (0, 50, 100, 150, 200 ng/ml) for 10 minutes at 4 °C. Merged images are shown.

Scale bar 10 µm.

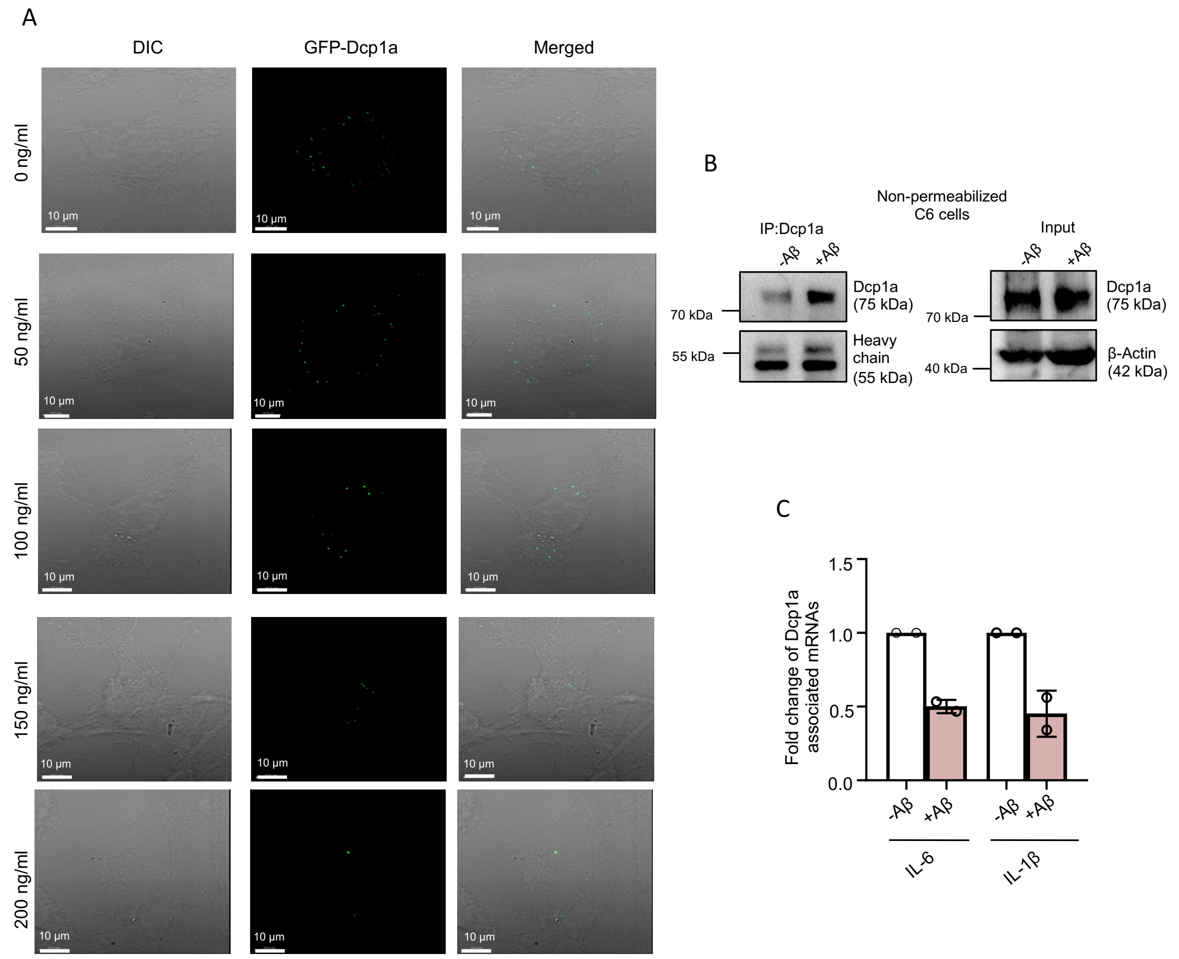

Figure S2 Digitonin permeabilization effects on internal P-bodies in C6 cells. Related to Figure 3.

(A) Confocal Images of GFP-Dcp1a (Green) expressing C6 cells permeabilized with increasing concentration of Digitonin (0, 50, 100, 150, 200 ng/ml) for 10 minutes at 4 °C. Merged images are shown. Scale bar 10 µm.

(B) (C) Immunoprecipitation of total endogenous Dcp1a protein from 0 or 2.5 µM A$\beta_{(1-42)}$ oligomer-treated C6 cells, without permeabilization. Western blot images of pulled-down Dcp1a level, along with heavy chain and input Dcp1a level with β-Actin as loading control, are shown. Molecular weight markers are also shown (B), followed by qRT-PCR-based quantification of IL-6 and IL-1β mRNA levels, normalized to relative pulled-down Dcp1a levels (n=2 independent experiments) (C).

Table S1: List of primary antibodies

Item	Raised in	Supplier	Catalogue No./RRID	Dilution for WB	Dilution for IF/IP
DDX6	Rabbit	Bethyl	A300-461A/ AB_2277216	1:10000	-
DCP1A	Mouse	Abnova	H0055802-M06	1:1000	1:100 /1:100
HA	Rat	Roche	11867423001/ AB_390918	1:1000	1:100 /1:100
β-Actin (HRP)	Mouse	Sigma	A3854-200UL/ AB_262011	1:10000	-
Myc	Mouse	Santa Cruz	(9E10):sc-40	1:1000	-
β-Tubulin	Mouse	Sigma-Aldrich	T5201/ AB_609915	1:6000	-
GAPDH	Mouse	Sigma-Aldrich	G9295/ AB_1078992	1:6000	-
eIF2C2	Mouse	Abnova	H00027161-M01/ AB_565459	-	1:100

Table S2: List of secondary antibodies

Item	Supplier	Catalogue No.	Dilution for WB	Dilution for IF
HRP Goat anti-rat	Invitrogen	62-9520	1:8000	-
HRP Goat anti-rabbit	Invitrogen	65-6120	1:8000	-
HRP Goat anti-mouse	Invitrogen	62-6520	1:8000	-
Alexa Fluor 568 (mouse)	Invitrogen	A11004	-	1:500
Alexa Fluor 488 (rat)	Invitrogen	A11006	-	1:500

Table S3: List of chemicals and reagents

Item	Supplier	Catalogue No.
Dulbecco's modified Eagle's medium	Gibco	12800-017
Heat-inactivated fetal bovine serum	Gibco	10082-147
Heat-inactivated horse serum	Gibco	16050-122
Penicillin-Streptomycin	Gibco	15140122
Nerve Growth Factor, 2.5S, Murine	Promega	G514A
Lipofectamine 2000 reagent	Invitrogen	11668-019
Trypsin-EDTA	Gibco	25200-072
1X PBS (pH=7.4)	Gibco	10010-023
Digitonin	Calbiochem	300410
Ultrapure nuclease-free water	Invitrogen	10977-015
Magnesium Chloride	Merck	M8266
Potassium Chloride	Merck	P9541
DTT	Roche	10197777001
PMSF	Omnipure	7110
Paraformaldehyde	Sigma	P6148-500G
Bovine serum albumine	Hi-media	mB083
Goat serum	Invitrogen	16210-064
Triton-X-100	Calbiochem	648468
Vectashield mounting medium with DAPI	Vector laboratories	(H-1500)
Sodium Bicarbonate	Merck	S5761
Glycine	Merck	G8898
Tris	Merck	93362
Hydrochloric Acid	Thermo Fisher Scientific	7647-01-0
10% SDS	Invitrogen	15553-027
Cy3	GE Healthcare	Q13108

Ethanol	Merck	K45117883346
Formamide	USB	75828-500G
Dextran Sulfate	Calbiochem	265152
Vanadyl Ribonucleoside Complex	Sigma	96740
Salmon Sperm DNA	Invitrogen	15632011
Amyloid Beta (1-42)	American Peptide	N/A
RNase Inhibitor	Thermo Fisher Scientific	N8080119
Sodium deoxycholate	Sigma	30970-100G
Protein G agarose beads	Thermo Fisher Scientific	20399
TRIzol LS reagent	Thermo Fisher Scientific	10296028
Chloroform	Sigma-Aldrich	C2432-500ML
Isopropanol	Sigma-Aldrich	278475-100ML
Glycoblue co-precipitant	Thermo Fisher Scientific	AM9516

Table S4: List of plasmids

**Name**	**Source/Reference**	**Description**
FLAG-HA-Ago2	Kind gift from Tom Tuschl	Plasmid expressing FLAG and HA tagged human Ago2
GFP-Dcp1a	Kind gift from Witold Filipowicz	Dcp1a cloned in frame with GFP
pCI-neo	Promega	pCI-neo expressing vector
Rheb-Myc	Kind gift from J. M. Backer	Myc tagged human Rheb expression plasmid
pDsRed2-ER	From Clonetech	ER targeting variant of DsRed
RL-IL-6	Prepared by cloning	3’UTR of IL-6 downstream of RL coding region in pCI-Neo vector
RL-IL-1β	Prepared by cloning	3’UTR of IL-1β downstream of RL coding region in pCI-Neo vector

Table S5: List of commercial assay kits

Item	Supplier	Catalogue No.
Reverse Transcriptase Core Kit	Eurogentec	RT-RTCK-03
MESA GREEN qPCR Master Mix Plus for SYBR Assay	Eurogentec	UF-FSMT-B0701

Table S6: List of cell lines

Item	Supplier	Catalogue No.
PC12	ATCC	CRL-1721
C6	ATCC	CCL-107

Table S7: List of oligonucleotides

Item	Supplier	Catalogue No.
Primers against ***GAP43*** target gene:<br><br>Forward-<br>5’ ACGAGAAGAAGGGTGATGCA 3’<br>Reverse-<br>5’CTTCGCCCTTCTTCTCCTCA 3’	This paper	N/A
Primers against ***Neurofilament-M*** target gene:<br><br>Forward-<br>5’ AACGTCAAGATGGCTCTGGA 3’<br>Reverse-<br>5’ GTGTTGGACCTTGAGCTTGG 3’	This paper	N/A
Primers against ***HuD*** target gene:<br><br>Forward-<br>5’ TCACCATTGACGGGATGACA 3’<br>Reverse-<br>5’ ACCTTGACGTTGTTCACTGC 3’	This paper	N/A
Primers against ***HuR*** target gene:<br><br>Forward-<br>5’ TCAACTCCAGGGTCCTTGTG 3’<br>Reverse-<br>5’ GTTCTGGTTGGGATTGGCTG 3’	This paper	N/A
Primers against ***IL-6*** target gene:	This paper	N/A

Forward-<br>5’ TACCCCAACTTCCAATGCTC 3’<br>Reverse-<br>5’ ACCACAGTGAGGAATGTCCA 3’		
Primers against ***IL-1β*** target gene:<br><br>Forward-<br>5’ GTGGATCCCAAACAATACCC 3’<br>Reverse-<br>5’ AACTATGTCCCGACCATTGC 3’	This paper	N/A
Primers against ***GAPDH*** target gene:<br><br>Forward-<br>5’ CAGGGGGGAGCCAAAAGGG 3’<br>Reverse-<br>5’ CTTGGCCAGGGGTGCTAAGC 3’	This paper	N/A
Oligo against ***RL*** sequence<br><br>5’ aT*cacaaagatgatT*ttctttggaaggtT*ca 3’<br>(Eurogentec)	This paper	N/A
Oligo against ***RL*** sequence<br><br>5’ aT*tagctggaggcagcgT*taccatgcagaaa 3’<br>(Eurogentec)	This paper	N/A
Oligo against ***RL*** sequence<br><br>5’ aT*agtccagcacgtT*catttgcttgcagT*g 3’<br>(Eurogentec)	This paper	N/A

Table S8: List of software

**Item**	**Supplier**	**RRID**
Imaris7	Bitplane	SCR_007370
Prism (v5.00 and v8.00)	GraphPad	SCR_002798
